# Analyzing the network structure of students’ motivation to learn AI: a self-determination theory perspective

**DOI:** 10.1038/s41539-025-00339-w

**Published:** 2025-07-27

**Authors:** Jiajing Li, Jianhua Zhang, Ching Sing Chai, Vivian W. Y. Lee, Xuesong Zhai, Xingwei Wang, Ronnel B. King

**Affiliations:** 1 College of Education for the Future, Beijing Normal University, Zhuhai, China; 2 Department of Curriculum and Instruction, Faculty of Education, The Chinese University of Hong Kong, Hong Kong, China; 3 Centre for Learning Enhancement And Research, The Chinese University of Hong Kong, Hong Kong, China; 4 College of Education, Zhejiang University, Hangzhou, China; 5 School of Environment, Tsinghua University, Beijing, China

**Keywords:** Education, Psychology

## Abstract

Motivation is a key driver of learning. Prior work on motivation has mostly focused on conventional learning contexts that did not necessarily involve AI. Hence, little is known about students’ motivation to learn AI. This study examined the structure of students’ AI motivational system using self-determination theory as the theoretical framework. Self-determination theory posits that there are qualitatively distinct types of motivation, including intrinsic motivation, identified regulation, introjected regulation, external regulation, and amotivation. Students' motivation, in turn, is strongly shaped by whether their basic psychological needs for competence, autonomy, and relatedness are satisfied. We used network analysis to explore the structure of students’ AI motivation. Participants included 1465 students from 47 universities. Introjected regulation was central to the AI motivational system but intrinsic motivation was less central. This meant that many students learned AI primarily out of guilt or shame and not because of personal enjoyment. Furthermore, competence satisfaction seemed more important than autonomy and relatedness satisfaction in AI-enriched learning environments. Hence, key practical implications include the need to have clear goals and standards as well as to build students' competence in using AI tools. This study enriches the AI education literature by focusing on students' motivational systems and suggesting ways to cultivate better engagement with AI.

## Introduction

Artificial intelligence (AI) is exerting a profound influence on teaching and learning^1–3^. It has great potential to empower teachers to personalize teaching practices and promote students’ learning outcomes^4–6^. AI technologies have been incorporated into teaching, learning, assessment, and administration^7^. Across the globe, nations are developing strategic plans to empower upcoming generations with the abilities necessary to thrive in the digital era^2,8^. Higher education institutions are prioritizing the integration of AI into the curriculum of university students, with the number of AI-related courses experiencing a substantial surge of 102.9% from 2018 to 2021^9^.

Due to the popularity of AI, research in this field has been flourishing. Previous studies have focused on the integration of AI into pedagogy and assessment or the development of AI-related educational policies^7,10–13^. However, students’ motivation to learn with AI has received less attention as much of the existing research on motivation has focused on traditional classroom settings which did not necessarily include AI-related components^14–16^. With AI being increasingly infused into classrooms, it is important to understand how motivation to learn AI could drive learning in such settings. This is a crucial issue as motivation is among the most powerful forces in learning^17–19^.

Hence, the goal of this study is to understand students’ AI motivation, which could complement existing research on motivation in conventional learning contexts. We used a network analysis approach to explore the structure of students’ motivational system, identify the most central factor of students’ AI motivation (i.e., the component most strongly connected to others), and explore how AI motivational factors are associated with other key correlates.

In this study, we used self-determination theory (SDT) as the overarching theoretical framework. SDT is one of the most widely applied motivation frameworks in education and psychology^20,21^. It is one of the most integrative motivation theories, underlining the multidimensionality (or quality) of motivation^22^. SDT has been used in thousands of studies across contexts such as education^23,24^, psychology^25^, health^26^, sports^27^, work^28^, and social relationships^29^.

A key tenet of SDT is that motivation is not just about quantity but also quality^21^. In the domain of AI education, quantity pertains to how much motivation they have to learn with AI, while quality pertains to their qualitatively distinct reasons for engaging with AI. Emphasizing both the quantity and quality of motivation makes it an ideal framework to examine students’ motivation to learn AI. Based on the degree of internalization, SDT differentiates intrinsic motivation, identified regulation, introjected regulation, external regulation, and amotivation^21^. According to SDT, the more internalized types of motivation are supposed to link to better learning outcomes^1^. Intrinsic motivation is the most internalized type, which refers to the engagement in learning activities out of interest, enjoyment, or internal satisfaction. Identified regulation denotes the behaviors driven by identifying the value of the activities. Introjected regulation in activities denotes engagement in learning in order to maintain self-esteem and avoid being recognized as an incapable person. External regulation pertains to behaviors propelled by external reasons such as punishments or rewards. Amotivation is a lack of intentionality^21^. This focus is particularly relevant given the unique characteristics of AI-assisted learning, which not only demand a significant amount of engagement but also require meaningful and sustained interactions with technology. The long-term benefits of high-quality motivation have been evidenced^14,15^. As suggested by previous research on AI-assisted teaching, initial excitement and enthusiasm about AI technologies might diminish over time (also called “novelty effect”). Some students might even be distracted by AI technologies over time. Hence, sustaining high quality motivation becomes a pressing topic.

Studies grounded in the SDT framework have extensively indicated that students are driven by different types of motivation^30^. In the domain of AI education, students might engage with AI for different reasons. Some students prioritize enjoyment or interest in learning AI (i.e., intrinsic motivation), while others may be primarily driven by shame and guilt (i.e., introjected regulation). Alternatively, some other students might recognize that it is important for one’s personal and professional development (i.e., identified regulation).

It is worth noting that students do not just exhibit one type of motivation; instead, they can have different types of motivation^21^. More importantly, students’ motivational orientations are not isolated from each other, they are intertwined in a motivational system^31,32^. For example, a student who attaches great importance to learning AI (identified regulation) may enjoy the process (intrinsic motivation), feel ashamed if they do not learn AI (introjected regulation), and be attracted by the rewards (external regulation) that AI can bring. According to researchers adopting the complex dynamic systems perspective, motivation involves a complex interplay of various interdependent motivational factors that collectively influence an individual’s learning experience. Change in any one factor could reverberate throughout the system. Hence, motivation should be studied as a whole system^33,34^. However, previous studies have primarily focused on how certain types of motivation to learn AI operate, with few of them accounting for the mutual interactions among these motivational factors. The current study sought to explore the motivational system underlying students’ AI learning by viewing the motivation to learn AI as a dynamic system in which different types of motivation interact with each other.

SDT foregrounds the roles of basic psychological needs satisfaction, social support, and facilitating conditions as catalysts for students’ motivation. These factors could also play a role in students’ motivation to learn AI. According to SDT, three basic psychological needs are manifested by the need for autonomy (i.e., feeling freedom in making choices), competence (i.e., feeling capable of performing tasks), and relatedness (i.e., feeling connected with others)^35^. SDT proposes that the fulfillment of students’ needs for autonomy, competence, and relatedness can lead to more adaptive types of motivation (e.g., identified regulation and intrinsic motivation). However, which among these three basic needs is primary remains an open question. Some prior studies have regarded autonomy need satisfaction as a primary target for intervention^36–38^. Still, some other researchers asserted competence need satisfaction surpassed autonomy and relatedness satisfaction as the strongest precursor of motivation^23,39^. For instance, Bureau et al. synthesized 144 studies covering over 79,000 students. They reported that competence need satisfaction outperformed autonomy and relatedness satisfaction in associating with intrinsic motivation and identified regulation. Hence, in this study, we examined these three basic psychological needs (autonomy, relatedness, and satisfaction) and what role they play in the students’ AI motivational system.

Apart from basic psychological needs satisfaction, SDT posits that environmental factors also play vital roles in shaping students’ motivation^40^. The theory underscores the importance of not only meeting individual psychological needs but also creating a supportive and resource-rich learning environment to foster students’ motivation. Hence, in the present study, we accounted for both environmental factors (i.e., social support and facilitating conditions) and basic psychological needs satisfaction.

Social support refers to the degree to which an individual perceives significant others support or encourage the use of AI^41,42^. It typically comes from peers, parents, and teachers and can also exert notable influences on how students interact with AI. Researchers found that an AI-supportive context could enhance students’ intrinsic motivation to learn AI^24,43^.

In addition, facilitating conditions, defined as the availability and accessibility of resources (e.g., technical support and access to AI applications) to facilitate learning with AI^41^, also have a role to play in relating to motivation to learn AI^44,45^. Those who can access more resources related to AI exhibited higher levels of intrinsic motivation^43,46^.

Overall, examining the connections between these correlates and AI motivational factors holds significant practical value, as understanding these relationships can guide the development of strategies to create a more engaging and motivating environment. Both SEM and network analysis could be leveraged to explore the complex interplay among variables related to AI motivation. They are designed to answer different types of research questions, which we elaborate below.

SEM is well suited to evaluate how well the proposed model fits the observed data and examine the extent to which the variance in the dependent variable can be explained by the independent variable. SEM typically examines the specific associations among latent constructs through correlations or linear regression, with the relationships among factors explicitly specified. In SEM models, diverse types of motivation were considered as separate indicators without synergies, as SEM assumes that each type of motivation operates independently from the others^47^.

Network analysis is an approach that complements SEM by providing an in-depth understanding of the most influential factors within the motivational system and allowing for a holistic view of students’ motivation. It has been increasingly applied to domains such as clinical psychology^48–50^, educational psychology^32,51,52^, and personality research^53^, among others.

On the one hand, network analysis conceptualizes constructs as interconnected systems of factors^54^. It echoes the assertion that there are complex structures and patterns of relationships among a constellation of factors in ecological settings^34^. More specifically, network analysis underscores that different components interact with each other and that some factors are more “central” and others are more “peripheral”^55^. Network analysis allows the comparisons of different networks, probing not only the overall structure and global strength but also the centrality of each variable and the strength of their connections^56^. With its capability to pinpoint and visualize central variables, network analysis could inform targets for successful interventions.

On the other hand, different from the SEM method that establishes a few direct paths between variables determined by researchers a *priori*, network analysis, as an exploratory approach, generates an undirected graph where edges are estimated between all nodes^57^. Nodes represent observed variables and are connected by edges that indicate statistical associations between them^58^. In particular, SEM requires the formulation of certain hypotheses and the specification of directional relationships between variables. For instance, we presume the paths should start from intrinsic motivation to extrinsic motivation and amotivation or should begin with amotivation and proceed to intrinsic and extrinsic motivation in carrying out SEM. In contrast, network analysis allows for the exploration of the data without predefined assumptions about the directionality of relationships. It could diagnose all possible connections between variables and are able to observe unexpected relationships. For example, network analysis could identify 10 distinct paths when there were five motivational factors, calculated as 5 * (5 -1)/2. These paths (e.g., intrinsic motivation → identified regulation, and identified regulation → intrinsic motivation) were considered simultaneously, with no specified directionality. Hence, network analysis could offer fine-grained insight into the interconnectedness among variables of interest. Employing network analysis to explore motivational factors holds promise in extending SDT findings to the domain of AI education^21,59^.

The identification of system components (network nodes) and the strength of the connection between nodes (network edges) are critical^60^. Network nodes are connected by network edges. To construct a network model, three steps can be followed: (1) network estimation: estimate a statistical model on data, from which some parameters can be represented as a weighted network between observed variables; (2) centrality estimation: analyze the weighted network structure; (3) accuracy and stability estimation: evaluate the accuracy of the network parameters and measures^58^. In the present study, we leveraged the network analysis to identify which motivational factors are most central to students’ AI motivational system and AI motivational system with correlates.

In light of the research gaps, this study aimed to (1) examine the most central component in the AI motivational system and (2) explore how basic needs satisfaction, social support, facilitating conditions, and AI motivation are interconnected in the AI motivational system. We used network analysis to answer these two questions. Given the widespread use of SEM, we also carried out supplementary analyses using this method. The results may be particularly relevant for those interested in the relationships among the constructs. The SEM model disentangled the nexus of different types of AI motivation (i.e., intrinsic motivation, identified regulation, introjected regulation, external regulation, and amotivation) and their correlates (i.e., basic psychological needs satisfaction, social support, and facilitating conditions).

## Results

Descriptive statistics including means, standard deviations, and Pearson correlation coefficients are presented in Supplementary Table 1. The distributional properties of each variable approximate a normal distribution, with skewness ranging from −0.74 to 0.91 and kurtosis ranging from −0.45 to 0.60^61^. Results of the correlation analysis indicated that all variables were correlated with each other with *r* ranging from −0.08 (*p* < 0.01) to 0.66 (*p* < 0.01), except for the correlations between external regulation and autonomy need satisfaction (*r* = 0.04, *p* > 0.05), and amotivation and relatedness need satisfaction (*r* = −0.03, *p* > 0.05) were non-significant.

To address our research aims, two models were developed. Model 1, the motivational system, examines the interrelationships among various types of students’ motivation to learn AI. Model 2, the motivational system with correlates, disentangles how these motivations are linked to correlates, including students’ satisfaction of basic psychological needs, social support, and facilitating conditions.

Model 1: Motivational system. In order to explore the interrelationships among different types of students’ motivation towards AI, intrinsic motivation, identified regulation, introjected regulation, external regulation, and amotivation were incorporated to construct the motivational system. Following the guidelines of Epskamp et al.^58^, the network analysis involved three steps: network estimation, centrality estimation, and accuracy and stability estimation.

### Network estimation

To discern the structure of the motivational system, we estimated a regularized network involving 5 features and 20 edges (5*[5–1]/2). Among 20 edges, 10 had non-zero weight (mean weight = 0.14).

### Centrality estimation

**Fig. 1:**
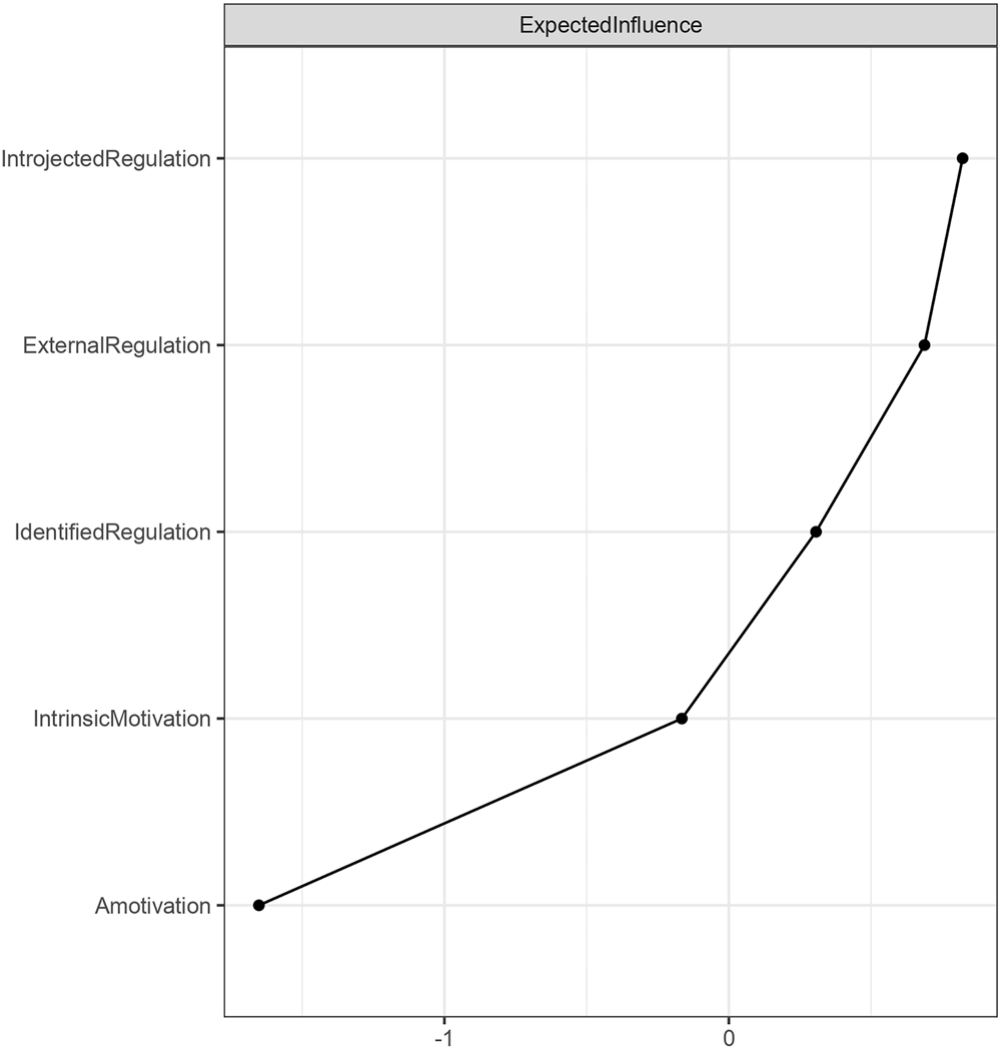
Standardized node centrality for AI motivation. This figure displays the standardized node centrality for AI motivation. Introjected regulation had the highest expected influence in the motivational system, followed by external regulation, identified regulation, intrinsic motivation, and amotivation.

**Fig. 2:**
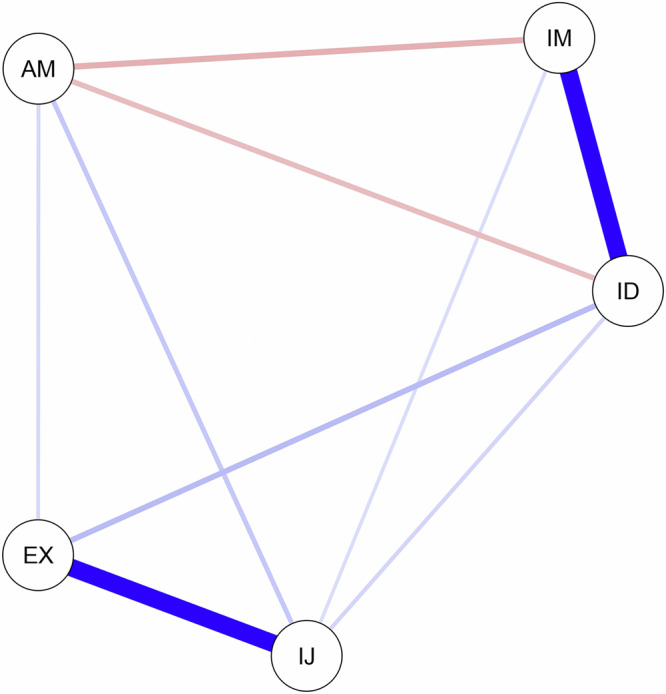
AI motivational system. This figure depicts the AI motivational system. Intrinsic motivation and identified regulation exhibited a strong association, as did introjected regulation and external regulation. IM intrinsic motivation, ID identified regulation, IJ introjected regulation, EX external regulation, AM amotivation. Edge thickness reflects the strength of association. The red edges represent negative relations. The blue edges represent positive relations.

Centrality indices for each feature in the motivational system are presented in Fig. 1. We found that introjected regulation had the highest expected influence. Hence, introjected regulation plays the most pivotal role in connecting and influencing other motivational factors^62^. Interestingly, we observed that the expected influence of intrinsic motivation ranked among the bottom two factors in the network, just above amotivation. In Fig. 2, the sizes of edges represent the strength between the two nodes. Intrinsic motivation and identified regulation exhibited a strong association, as did introjected regulation and external regulation.

### Accuracy and stability estimation

Supplementary Figs. 1–3 suggest that the AI motivational network was accurately estimated. The correlation stability coefficient (CS coefficient) is an indicator of the stability of centrality indices. It provides information about the consistency of centrality measures across different samples^63^. Using case-dropping bootstrap analysis, where progressively fewer cases were sampled from the original data set to obtain random subsamples (retaining only a certain portion of cases; from 90% to 10%). The CS coefficient for strength and expected influence was 0.75.

Model 2: Motivational system with correlates. After analyzing how the multiple types of motivation work together, we then simultaneously added key correlates including the satisfaction of the basic psychological needs for autonomy, competence, and relatedness as well as social support and facilitating conditions. Again, we followed the three-step guidelines outlined by Epskamp and colleagues^58^.

### Network estimation

For the connections between the motivational system and its correlates, we estimated a regularized network involving 10 features and 45 edges (10*[10–1]/2). Among 45 edges, 35 had non-zero weight (mean weight = 0.07).

### Centrality estimation

**Fig. 3:**
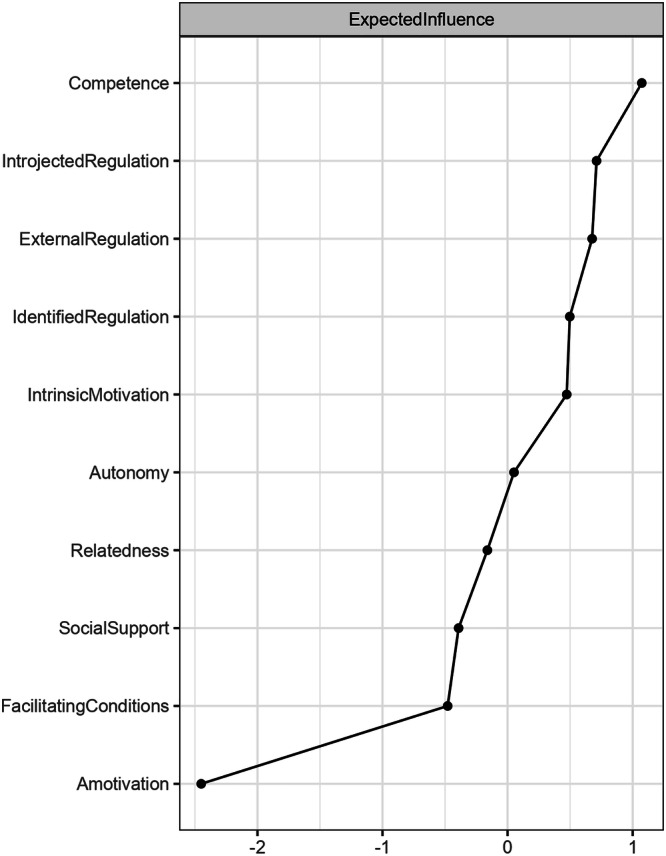
Standardized node centrality for AI motivation and correlates. This figure shows the standardized node centrality for AI motivation and correlates. Competence need satisfaction emerged as the predictor with the highest expected influence value among the four correlates.

**Fig. 4:**
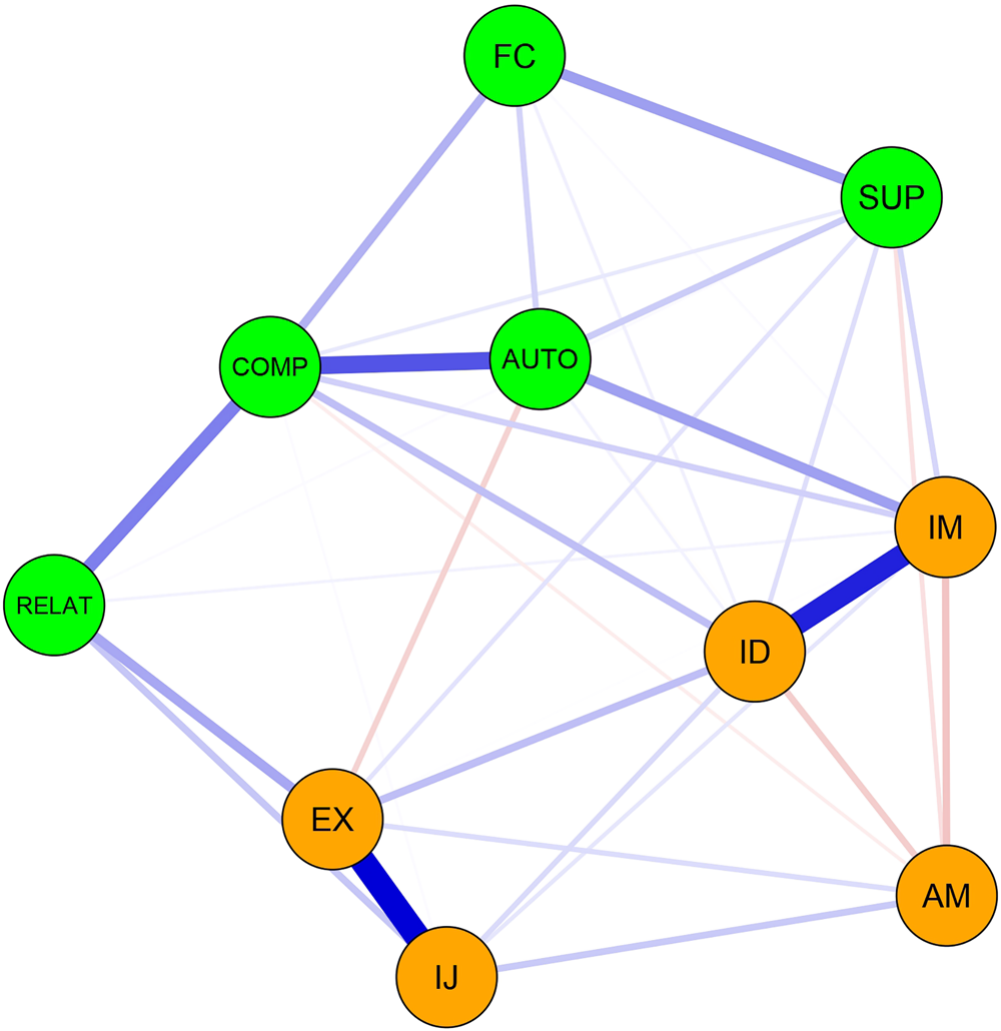
AI motivational system and correlates. This figure represents the AI motivational system and correlates. Intrinsic motivation was strongly associated with identified regulation, while introjected regulation was closely linked with external regulation and autonomy need satisfaction was strongly related to competence need satisfaction. IM intrinsic motivation, ID identified regulation, IJ introjected regulation, EX external regulation, AM amotivation, AUTO autonomy satisfaction, COMP competence satisfaction, RELAT relatedness satisfaction, SUP social support, FC facilitating conditions. The green nodes represent the correlates of AI motivation, while the orange nodes represent AI motivation. The red edges indicate negative relations, and the blue edges indicate positive relations.

As depicted in Fig. 3, competence need satisfaction emerged as the predictor with the highest expected influence value. Therefore, it exhibited higher centrality and importance within the motivational system. Additionally, the expected influence of intrinsic motivation still ranked among the bottom factors in the network, just above amotivation. Figure 4 shows the strength of the connections between nodes. A strong relationship was observed between intrinsic motivation and identified regulation, introjected regulation and external regulation, and the satisfaction of autonomy and competence needs.

### Accuracy and stability estimation

Supplementary Figs. 4–6 suggested the accurate estimation of the motivation network with correlates. The CS coefficient for strength and expected influence was 0.75.

Given that most researchers are more familiar with CFA and SEM, we also included a supplementary analysis using CFA and SEM. The CFA model examined the construct validity of the responses to the scale (Supplementary Figs. 7, 8), while the SEM model theoretical linkages among five types of motivation and basic psychological needs satisfaction, social support, and facilitating conditions (Supplementary Fig. 9). Findings suggest that different types of motivation were significantly associated with these correlates.

## Discussion

In the current study, we mapped out the motivational system by modeling the network structure of students’ motivation to learn AI and investigating how they were associated with the social context. This study has two major findings: (1) introjected regulation is central (with the highest expected influence) to the AI motivational system and (2) competence need satisfaction was the most powerful correlate associated with AI motivation. The study offers a nuanced understanding of the AI motivational system.

Surprisingly, introjected regulation was the primary driver for students’ AI learning. We found this counterintuitive given that much of the work on SDT and the motivational literature has generally touted the benefits of more internalized types of motivation such as intrinsic and identified motivation^23,59^. However, in our study, introjected regulation was more salient compared to these other types of motivation. This means that students engaged in AI learning mainly because they felt it was expected of them by others. The leading role of introjected regulation seems to contradict the propositions of SDT which states that intrinsic motivation and identified regulation are central motivational factors and should be given more attention^21^.

A plausible explanation for the significance of introjected regulation might be the widespread integration of AI and the global surge of interest in the field. Universities and even entire nations recognize the potential of AI education and expect students to acquire AI knowledge and skills, leading to the desire to meet societal expectations^64,65^. With a growing demand for learning AI, students may internalize these AI learning expectations and engage in AI learning to avoid feeling guilty.

Another potential explanation for the centrality of introjected regulation is the high self-worth maintenance of Chinese students, who place great importance on self-image and preserving their ‘face’ in front of others^66–68^. They fear ‘losing face’, as acknowledging a lack of engagement in AI learning may be perceived as a sign of incompetence or falling behind. This fear is deeply rooted in cultural values that emphasize collective identity. Consequently, the concerns for preserving one’s self-image could drive students to prioritize introjected regulation in AI learning, as they want to get approval and avoid negative judgments from peers and significant others. Furthermore, the competitive atmosphere in China can exacerbate the need for validation, pushing students to engage in AI learning behaviors that align more with external expectations rather than intrinsic interests.

Intrinsic motivation or identified regulation was not identified as the primary driving force in the motivational system of AI learning. While intrinsic motivation and identified regulation are posited as the most adaptive types of motivation due to their alignment with personal satisfaction and long-term engagement^21^, they might be relatively ‘idealized’ scenarios in AI learning. Specifically, intrinsic motivation and identified regulation in AI learning are not always the most attainable types compared to other forms of motivation. Perhaps, the need for constant updating of knowledge to stay abreast of the rapid technological advancement may make intrinsic motivation and identified regulation harder to sustain^69,70^. This sheds light on the significant differences between AI education and conventional classroom learning, highlighting the need for special attention to unique aspects of AI learning. Educators may need to focus on cultivating students’ intrinsic and identified motivation towards AI learning and strengthen these more adaptive forms of motivation compared to the less adaptive introjected type of motivation.

Within the network, intrinsic motivation was among the factors with the lowest centrality, just above amotivation. Therefore, greater attention is particularly needed on intrinsic motivation, as it holds the potential to yield more desirable learning outcomes. By giving due attention to fostering intrinsic motivation, educators can empower students to become self-driven learners, thereby resulting in better AI literacy and maximizing the benefits of AI in their learning journey.

Motivational factors were contingent on basic psychological needs satisfaction, social support, and facilitating conditions. Competence need satisfaction was the most salient factor tied to motivation to learn AI. This supports some previous literature documenting that students’ need for competence exerts a stronger impact on motivation than their need for autonomy and relatedness^23,39^. The influential role of competence needs satisfaction brings fresh insight to the bulk of research revolving around autonomy-supportive teaching, thereby directing future endeavors on fulfilling competence needs in the field of AI education. This finding might stem from the high demands involved in AI learning. With the rapid pace of technological advancements, students are increasingly required to master AI skills. As a result, support for competence enables them to feel capable of acquiring advanced knowledge, thereby fueling their motivation for continuous effort^48,71^. Furthermore, the prevalent utilitarian-oriented climate in universities may also contribute to this finding^72,73^. Equipping students with AI skills and knowledge can enhance their productivity and problem-solving capabilities, hence, they are motivated to learn AI. This is a notable contribution to the prior works on AI learning based on SDT which predominantly assumed the crucial role of autonomy need satisfaction and paid greater attention to autonomy-supportive teaching.

Taken together, this study portrays a comprehensive picture of the mechanisms of motivation in the context of university students’ AI learning. Our findings underscore that students’ motivation to learn AI was primarily fueled by their desire to avoid feelings of guilt if they failed to do so. We also found that students were more likely to be motivated to pursue AI learning when they felt proficient in mastering AI.

The study has several caveats. First, the study is exploratory in nature. Future confirmatory studies that could unpack key theoretical mechanisms are needed to develop a more nuanced understanding of students' AI motivation. Second, the data were collected at a one-time point, hence, the longitudinal stability of the network and causal directionality cannot be generated. Future researchers may benefit from longitudinally navigating students’ motivation to learn AI. Third, participants in our study were all Chinese students, which might also explain why introjected regulation was the most central element in the network. Researchers are recommended to invite participants from diverse backgrounds around the world to enhance the generalizability of our findings. Fourth, heavy reliance on self-report surveys might lead to social desirability biases. Future studies may consider incorporating other measures such as semi-structured interviews to cross-validate the findings obtained.

Despite the above-mentioned limitations, our research has theoretical, methodological, and pedagogical implications. Theoretically, the findings of the present study enrich SDT motivational theorizing which has mostly been conducted in traditional classroom settings. Our work shows that SDT is also a suitable framework for understanding students’ motivation to learn AI^74,75^. Given the increasing prevalence of AI in the educational context, it is important for researchers to understand the reasons behind why students engage with AI or not in learning. Hence, it is crucial to examine the motivational underpinnings of AI learning.

Another significant theoretical contribution of our study is the notable influence of introjected regulation. By juxtaposing distinct types of AI motivation, we found the introjected regulation in AI learning surpassed other types of motivation and emerged as the most crucial factor of the motivational system. This nuanced perspective extends SDT, which asserts that intrinsic motivation and identified regulation are the cores. Vansteenkiste^76^ claimed that ‘the quality of motivation matters’, with intrinsic motivation and identified regulation being key indicators for motivational quality. However, the centrality of introjected regulation warrants future research attention.

Furthermore, prior work on SDT has mostly surfaced the importance of autonomy satisfaction. In fact, a quick look at the SDT literature would indicate that autonomy satisfaction has garnered the lion’s share of attention in the SDT literature^36–38^. However, our study illustrated competence satisfaction might be even more important at least in the context of AI motivation. This refines SDT, which did not make specific propositions about whether each need contributes equally or whether some are more relevant than others^23^.

Methodologically, this study is among the first to probe into the mechanism of different types of motivation and correlates in the realm of AI learning from a holistic network perspective. Although SDT has also been used in AI-infused learning contexts^47,48,57,74,75^, prior motivational research in the AI context has mostly relied on SEM techniques, which are not well-suited to understanding the AI motivational system. According to SDT, different types of motivation are intertwined to constitute a motivational system. Capitalizing on the network analysis, the study addresses the methodological shortcomings of previous studies. Firstly, the network analysis approach enables us to view students’ motivation to learn AI as a complex phenomenon wherein multiple motivations are interconnected with each other. Secondly, network analysis allows for a holistic understanding of the motivational system, estimating edges between all nodes. The study also attests to the applicability of network approaches in AI learning and could serve as a paradigm for future research with similar objectives.

Pedagogically, the study informs the intervention targets for promoting students’ motivation to learn AI. On the one hand, our findings highlight that introjected regulation was of paramount importance in the AI motivational system. However, introjected regulation is not the most optimal form of motivation. Therefore, educators are encouraged to implement strategies that help students nurture their identified and intrinsic motivation. To achieve this, they can help students understand the value of AI learning by incorporating AI-related content into the curriculum design, organizing AI-related activities, running workshops, and introducing real-world applications of AI^31^. Specifically, educators can incorporate topics such as natural language processing, machine learning, ethical considerations of AI, and affective computing into subjects such as language learning, science, social, and psychological studies^24,77,78^. To further motivate students, activities such as AI knowledge competitions, project-based AI learning, AI-powered games, and AI ethics debates can be organized. Regular workshops are also recommended to provide students with hands-on experience and equip them with essential AI knowledge and skills. Finally, by introducing real-world applications such as personalized learning systems and virtual interview assistants, educators can showcase how AI can be used to enhance effectiveness and decision-making. Governments and policymakers may consider initiating AI education and advocating how AI learning could increase the graduates’ competitiveness in the job market. Publicizing annual job reports that underline the significance of AI in meeting societal demands could be a feasible route. Moreover, more funding should be allocated to AI education, as it can directly influence the technological infrastructure and resources approachable by students and educators. Researchers could also work with the teaching and learning centers that are responsible for training and equipping university instructors with the necessary AI skills and upgrading their AI proficiency ^79^.

The network analysis results also suggest that building students' competence in AI-enriched environments might be more critical than giving them a sense of autonomy or fulfilling their relatedness needs. As a novel product of the digital age, AI could make some students feel insecure the future job market. Tertiary educators are suggested to pay close attention to students’ competence beliefs, fostering their sense of agency in controlling human-AI interactions and enhancing their confidence in leveraging AI to benefit their learning process^80^. To satisfy students’ basic psychological needs for competence, educators may consider choosing appropriate AI tools, providing clear guidance, establishing role models, and offering timely and frequent encouragement. In particular, the selection of chatbots based on students’ competencies is preferable^81^. Clear guidance is helpful in effectively navigating challenges and gaining a stronger sense of accomplishment as they progress^7^. Role models also play a crucial role in shaping students’ competence beliefs and motivation to learn AI^82^. Observing peers who are proficient in AI can enhance an individual’s belief in their own competence and increase their effort^43^. Educators can establish role models and encourage them to share ideas in classes. Timely and frequent encouragement from educators could also boost students’ motivation to master more AI knowledge.

Taken together, this study demonstrated the network structure of students’ motivation to learn AI. It enriches existing research on motivation which has mostly been explored in traditional classroom settings that did not necessarily involve AI. Motivation to learn AI is important in an educational context where learning with and about AI is becoming more prevalent and where the future world of work requires individuals to have basic AI literacy.

## Methods

The study was conducted in accordance with the Declaration of Helsinki and ethical standrads. The study received the ethical approval from the Chinese University of Hong Kong (approval no.: SBRE-23-0083). All participants signed a consent form prior to their involvement in the study, including an overview, the purpose, and confidentiality of the research.

Convenience sampling method was adopted to recruit participants. A total of 1465 university students were recruited from 47 universities in Greater China, including Mainland China and Hong Kong SAR. Of them, 460 and 1005 were from mainland China and Hong Kong SAR, respectively. As for majors, 820 were enrolled in non-STEM majors (i.e., arts, business administration, education, law, and social science), and 645 were from STEM majors (i.e., engineering, medicine, and science). Their mean age was 19.00 (*SD* = 18.42) and there were 848 (57.9%) female students.

Students rated on 7-point Likert scales with 1 indicating ‘*strongly disagree*’ and 7 indicating ‘*strongly agree*’. The original versions of the questionnaires are in English, we organized a committee to back-translate items. Both Chinese and English versions were offered to cater to students’ preferences. The details of measures are reported in the Table 1 and the full instruments are included in the Supplementary Table 2.

**Table 1:** Variable description

	Variable definition	Sample item
Correlates		
•Autonomy	Feeling freedom in making choices	I feel like I can make a lot of input in deciding how I use AI in learning
•Competence	Feeling capable of performing tasks	I think I am pretty good at learning with AI
•Relatedness	Feeling connected with others	When I learn with AI, I feel connected with my classmates
•Social support	Resources that individuals receive from their social networks	My teachers allow us to use AI for learning
•Facilitating conditions	The availability and accessibility of resources	I can easily find help when I need to know more about AI technology
AI motivation		
•Intrinsic motivation	Engagement in learning activities out of interest, enjoyment, or internal satisfaction	Learning with AI is fun for me
•Identified regulation	The behaviors driven by identifying the value of the activities	Learning with AI is important for my future career
•Introjected regulation	Engagement in learning in order to maintain self-esteem and avoid guilt and shame	I feel a sense of obligation to learn with AI because others expect it of me
•External regulation	The behaviors propelled by external reasons such as punishments or rewards	I will get in trouble if I don’t learn with AI
•Amotivation	A lack of intentionality and absence of motivation	I cannot come to see why I need to learn with AI

To gauge students’ motivation to learn AI, we employed the AI Motivation Scale by Li et al.^83^. It is comprised of intrinsic motivation (4 items; e.g., Learning with AI is fun for me), identified regulation (4 items; e.g., Learning with AI is important for my future career), introjected regulation (4 items; e.g., I feel a sense of obligation to learn with AI because others expect it of me), external regulation (4 items; e.g., I’m required to learning with AI), and amotivation (4 items; e.g., I cannot come to see why I need to learn with AI). The internal consistency of students’ responses to the five subscales ranged from 0.86 to 0.95.

Students’ basic psychological needs satisfaction was assessed using a scale by Xia et al.^57^. Perceived autonomy satisfaction (3 items; e.g., I feel like I can make a lot of input in deciding how I use AI in learning), perceived competence satisfaction (3 items; I think I am pretty good at learning with AI), and perceived relatedness satisfaction (3 items; When I learn with AI, I feel valued) were measured. Cronbach’s alpha coefficient for students’ responses to the scale was 0.81.

To measure students perceived social support, four items by Wang et al.^43^ were employed. A sample item was: “My teachers allow us to use AI for learning”. The internal consistency of the scale was 0.78.

Facilitating conditions were measured with 4 items adapted from Wang et al.^43^. A sample item was “I can easily find help when I need to know more about AI technology”. The internal consistency of the scale was 0.87.

SPSS 25.0 was used to conduct the preliminary analysis. We computed the descriptive statistics (i.e., means and standard deviations). To ensure the normality of distribution, skewness, and kurtosis were computed. If kurtosis and skewness fall within the range of ±2, the distributional properties of each variable approximate a normal distribution^61^. Cronbach’s alpha coefficients were employed to check the extent to which students’ responses to the scales were consistent. Pearson correlation coefficients were utilized to measure whether the measures are correlated.

After the preliminary analysis, we proceed to the network analysis using RStudio 1.2.5033 (R version 3.6.3). Two network models were tested: model 1 (motivational system) and model 2 (motivational system with correlates). The network analysis procedures follow the standard guidelines by Epskamp et al.^58^. It involves estimations of network, centrality, accuracy, and stability.

### Network estimation

The EBICglasso function of the graph package was employed. The network estimation procedure requires an estimate of the correlation matrix and returns a parsimonious network. The structure contains nodes and edges that represent different variables and the relations between variables, respectively^84^. The nodes in the network are composite scores, which are frequently applied in previous studies^85,86^. Different measures were gathered or computed for each variable including structural network metrics which were computed for evaluating the importance of individual variables and their relations. Overall network metrics were also computed.

### Centrality estimation

The centralityPlot function in the graph package was utilized in this step. We opt for the expected influence value to quantify the centrality or relative importance of each node in the network. The expected influence of a node was weighted by the absolute magnitude of its relevant edges, encompassing both positive and negative values^87^. A greater node expected influence indicates higher centrality and significance of the node within the network^88^. We chose expected influence metrics for two reasons. First, our research question mainly concerns identifying the most pivotal nodes^89^, rather than identifying nodes that bridge different clusters^49^. Second, our motivational system could include both positive and negative edges. For example, amotivation might be negatively related to other motivational factors. As outlined by previous researchers, expected influence has demonstrated superior performance compared to other types of centrality measures in networks with diverse types of edges^62^.

### Accuracy and stability estimation

We relied on the *bootnet* package to estimate the accuracy and stability of the network. Three steps were performed. First, bootstrapping was utilized to establish the 95% confidence intervals (CIs) of the edge weights. The narrower CIs or the less overlap between lower- and upper-level CIs denote lower sampling variability and higher accuracy of the estimated network. Second, the CS coefficient was calculated to identify the stability of centrality indices. A value above 0.25 and 0.50 indicates acceptable and excellent network stability, respectively^58^. Third, we compared the nodes’ centrality indices with bootstrapped difference testing to evaluate whether there are any significant discrepancies between them.

Although intrinsic motivation, which is the most “internalized” form of motivation, it does not necessarily mean that it is the most central factor in the motivational system^59^. Some researchers claimed that the type of motivation most closely associated with the outcome variable depends on the particular outcome being measured^27,30,59^. For example, Howard et al. found that performance goals were more closely associated with introjected regulation (*ρ* = 0.46 for performance approach goals and *ρ* = 0.43 for performance avoidance goals) than with intrinsic motivation (*ρ* = 0.25 for performance approach goals and *ρ* = 0.20 for performance avoidance goals). Additionally, persistence demonstrated the strongest relationship with identified regulation compared to other forms of motivation.

In addition, culture may also play a significant role^90,91^. In Eastern cultures, rather than being driven by pure intrinsic interests, some students are likely to pursue academic success to meet parental expectations or societal norms. As a result, they would devote more effort to their studies, often prioritizing academic performance over personal interest. In this case, their extrinsic motivation does not necessarily lead to maladaptive outcomes^92^.

## Supplementary information

Supplementary materials

## Data Availability

The datasets used and/or analyzed during the current study are available from the corresponding author on reasonable request.

## Code availability

The code for data analysis used in the current study is available upon request. Please contact the corresponding author. R version 3.6.3 was used to perform the main analysis.
