# Supplementary material for: Analyzing the network structure of students’ motivation to learn AI: a self-determination theory perspective
Source: NPJ Sci Learn. 2025 Jul 27;10:48. doi: 10.1038/s41539-025-00339-w (PMC12301463; doi:10.1038/s41539-025-00339-w)
Supplement: Supplementary file 1 — Supplementary materials [file 41539_2025_339_MOESM1_ESM.docx]

**Supplementary Materials**

**Section A**

Supplementary Table 1. Descriptive statistics and Pearson correlations among the measures

|  | 1 | 2 | 3 | 4 | 5 | 6 | 7 | 8 | 9 | 10 |
| --- | --- | --- | --- | --- | --- | --- | --- | --- | --- | --- |
| **Motivation** |  |  |  |  |  |  |  |  |  |  |
| 1. Intrinsic motivation | -- |  |  |  |  |  |  |  |  |  |
| 2. Identified regulation | .66** | -- |  |  |  |  |  |  |  |  |
| 3. Introjected regulation | .27** | .33** | -- |  |  |  |  |  |  |  |
| 4. External regulation | .25** | .34** | .65** | -- |  |  |  |  |  |  |
| 5. Amotivation | -.28** | -.25** | .12** | .11** | -- |  |  |  |  |  |
| **Correlates** |  |  |  |  |  |  |  |  |  |  |
| 6. Autonomy need satisfaction | .47** | .39** | .10** | .04 | -.19** | -- |  |  |  |  |
| 7. Competence need satisfaction | .48** | .47** | .23** | .18** | -.20** | .56** | -- |  |  |  |
| 8. Relatedness need satisfaction | .29** | .30** | .37** | .39** | -.03 | .22** | .42** | -- |  |  |
| 9. Social support | .36** | .34** | .16** | .19** | -.18** | .34** | .34** | .20** | -- |  |
| 10. Facilitating conditions | .28** | .27** | .10** | .10** | -.12** | .33** | .37** | .18** | .35** | -- |
| Mean | 5.00 | 5.10 | 3.19 | 3.17 | 2.82 | 3.88 | 4.65 | 4.89 | 5.15 | 4.90 |
| *SD* | 1.20 | 1.18 | 1.37 | 1.40 | 1.53 | 1.36 | 1.09 | 1.15 | 1.11 | 1.10 |
| Skewness | -.56 | -.74 | .48 | .38 | .91 | -.61 | -.35 | -.02 | -.23 | -.41 |
| Kurtosis | .34 | .60 | -.23 | -.40 | .09 | .43 | .32 | -.28 | -.03 | -.01 |
| Cronbach’s alpha | .91 | .86 | .87 | .86 | .95 | .89 | .78 | .87 | .83 | .80 |

*Note*. ** *p* < .01, * *p* < .05.

**Section B**

[Supplementary Figure 1. Edge-weight accuracy for AI motivation 2](#_Toc187073068)

[Supplementary Figure 2. Node strength centrality difference test for AI motivation 3](#_Toc187073069)

[Supplementary Figure 3. Centrality stability for AI motivation 4](#_Toc187073070)

[Supplementary Figure 4. Edge-weight accuracy for AI motivation and correlates 5](#_Toc187073071)

[Supplementary Figure 5. Node strength centrality difference test for AI motivation and correlates 6](#_Toc187073072)

[Supplementary Figure 6. Centrality stability for AI motivation and correlates 7](#_Toc187073073)

Supplementary Figure 1. Edge-weight accuracy for AI motivation


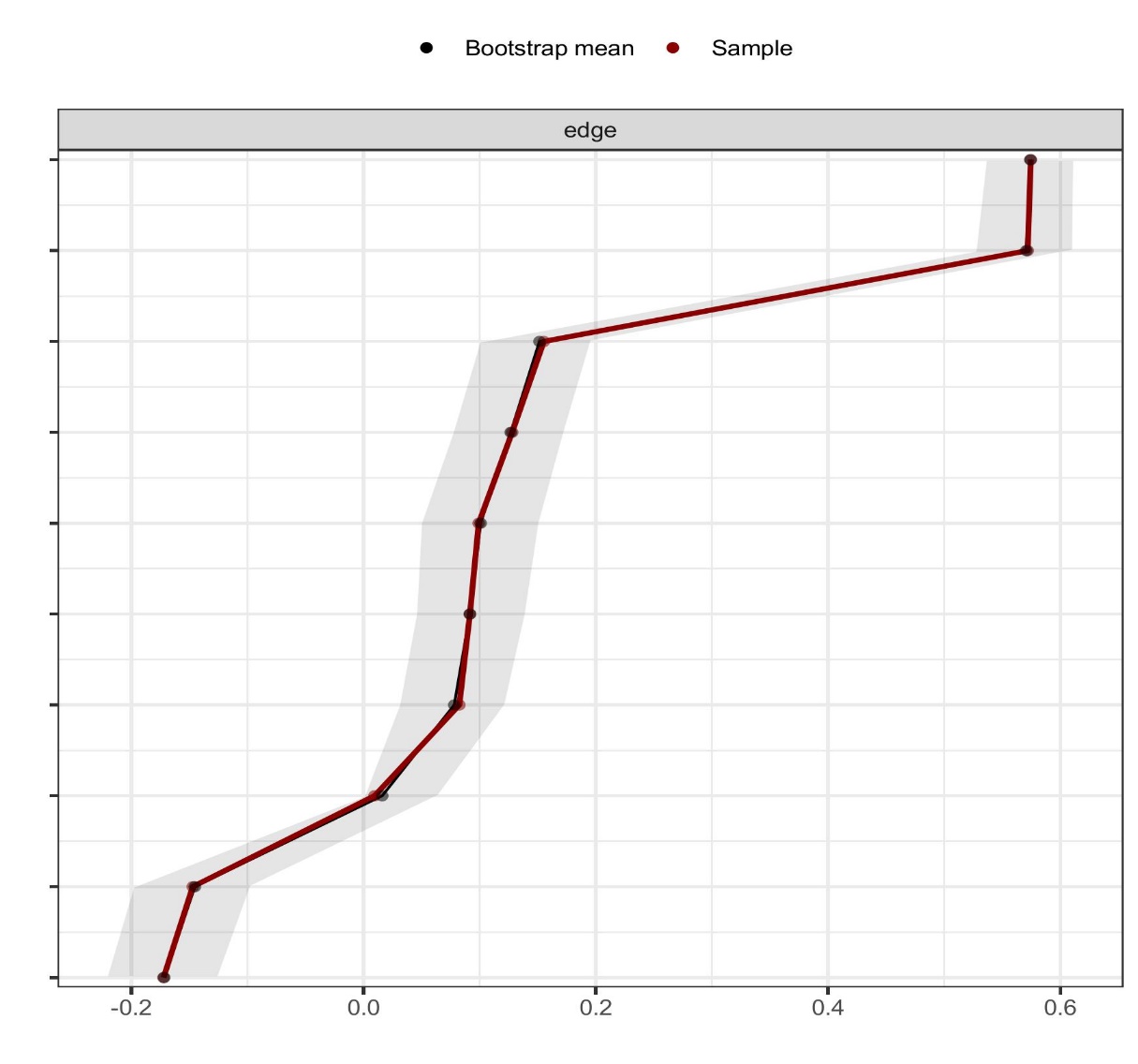


*Note.* This figure shows the edge-weight accuracy for AI motivation. The red line represents the sample value. The gray indicates the 95% confidence intervals. Each horizontal line represents one edge of the network, ordered from the edge with the highest edge-weight to the edge with the lowest edge-weight.

Supplementary Figure 2. Node strength centrality difference test for AI motivation


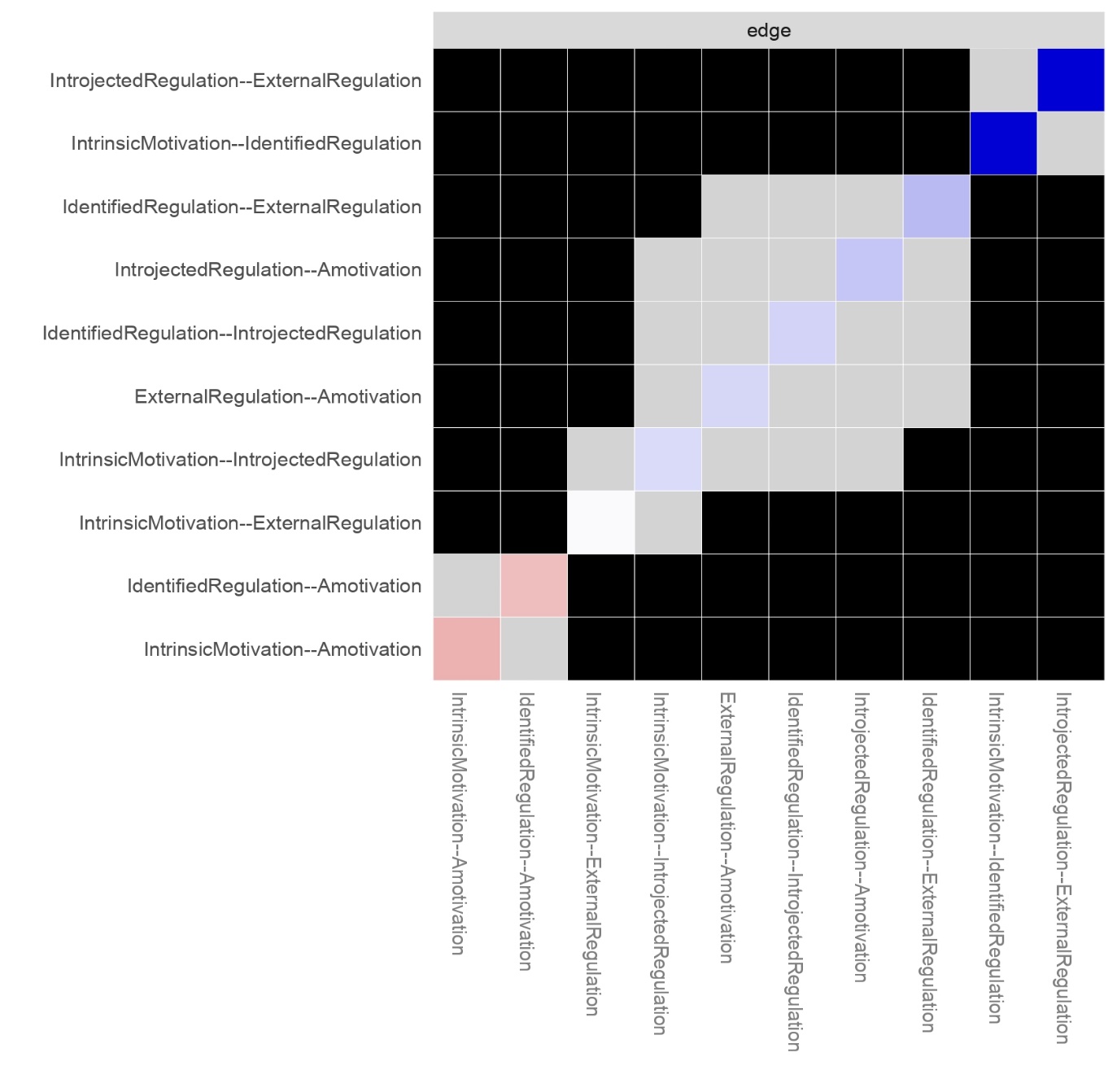


*Note.* This figure shows the node strength centrality difference for AI motivation. Black boxes denote nodes or edges that significantly differ from one another, while gray boxes indicate nodes or edges that do not, and the white box shows the value of node strength.

Supplementary Figure 3. Centrality stability for AI motivation


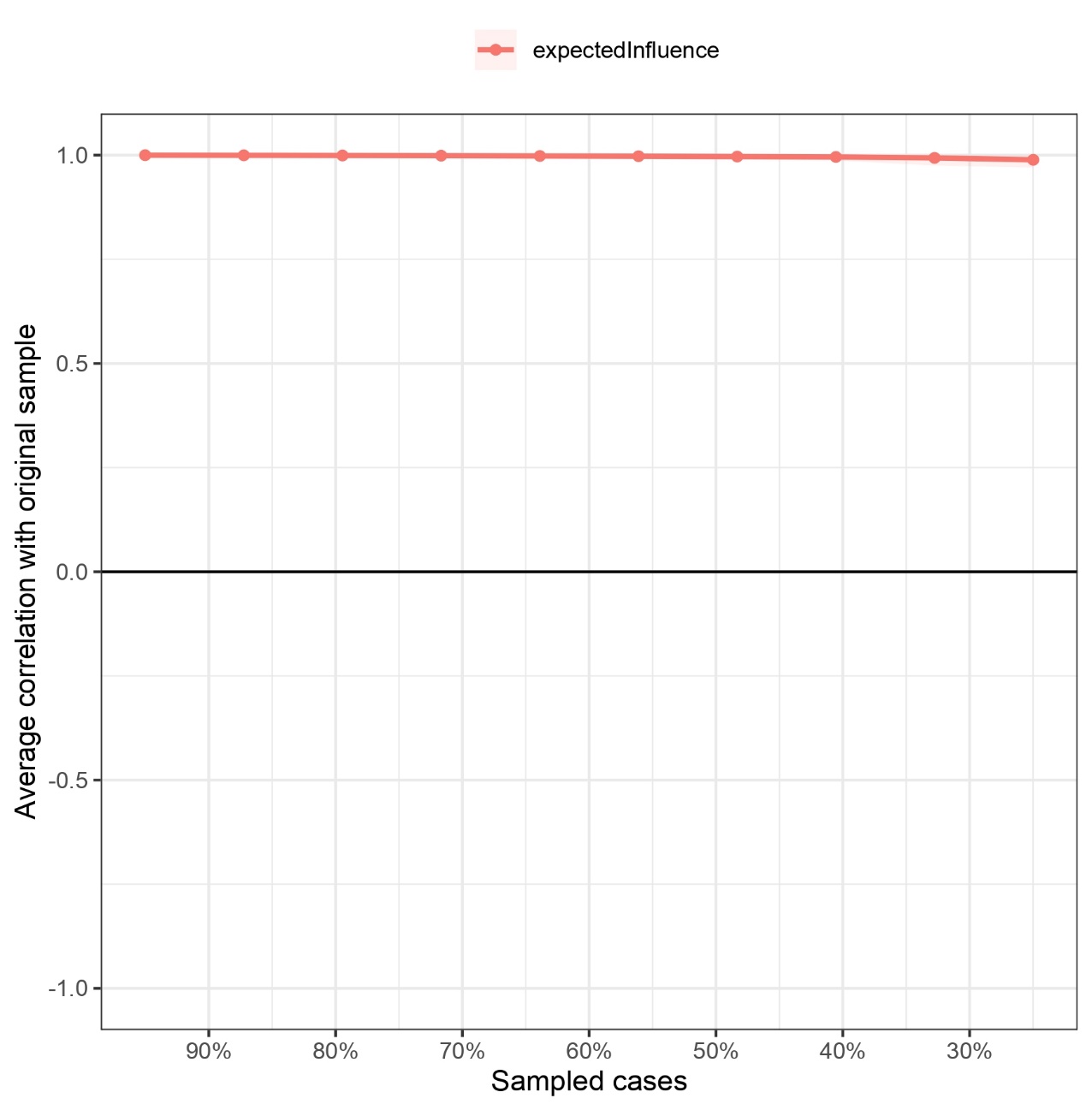


*Note.* This figure depicts the centrality stability for AI motivation. The x-axis illustrates the sample decrease from 95% to 25% of the original sample, and the y-axis illustrates the changes in correlation estimates between the subsample and the original entire sample. Lines indicate the means, and areas indicate the range from the 2.5th quantile to the 97.5th quantile.

Supplementary Figure 4. Edge-weight accuracy for AI motivation and correlates

**
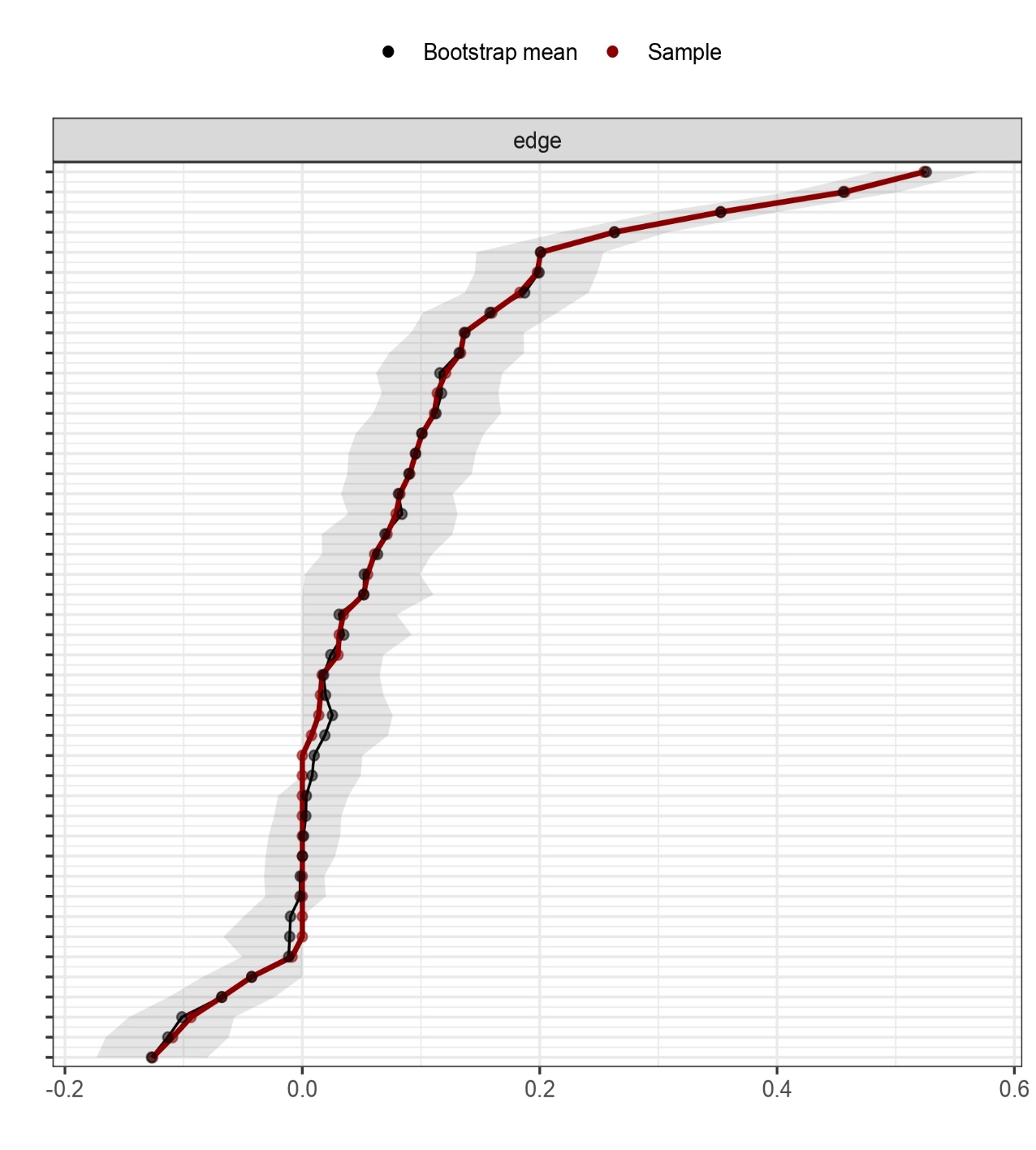
**

*Note.* This figure shows the edge-weight accuracy for AI motivation and correlates. The red line represents the sample value. The gray indicates the 95% confidence intervals. Each horizontal line represents one edge of the network, ordered from the edge with the highest edge-weight to the edge with the lowest edge-weight.

Supplementary Figure 5. Node strength centrality difference test for AI motivation and correlates


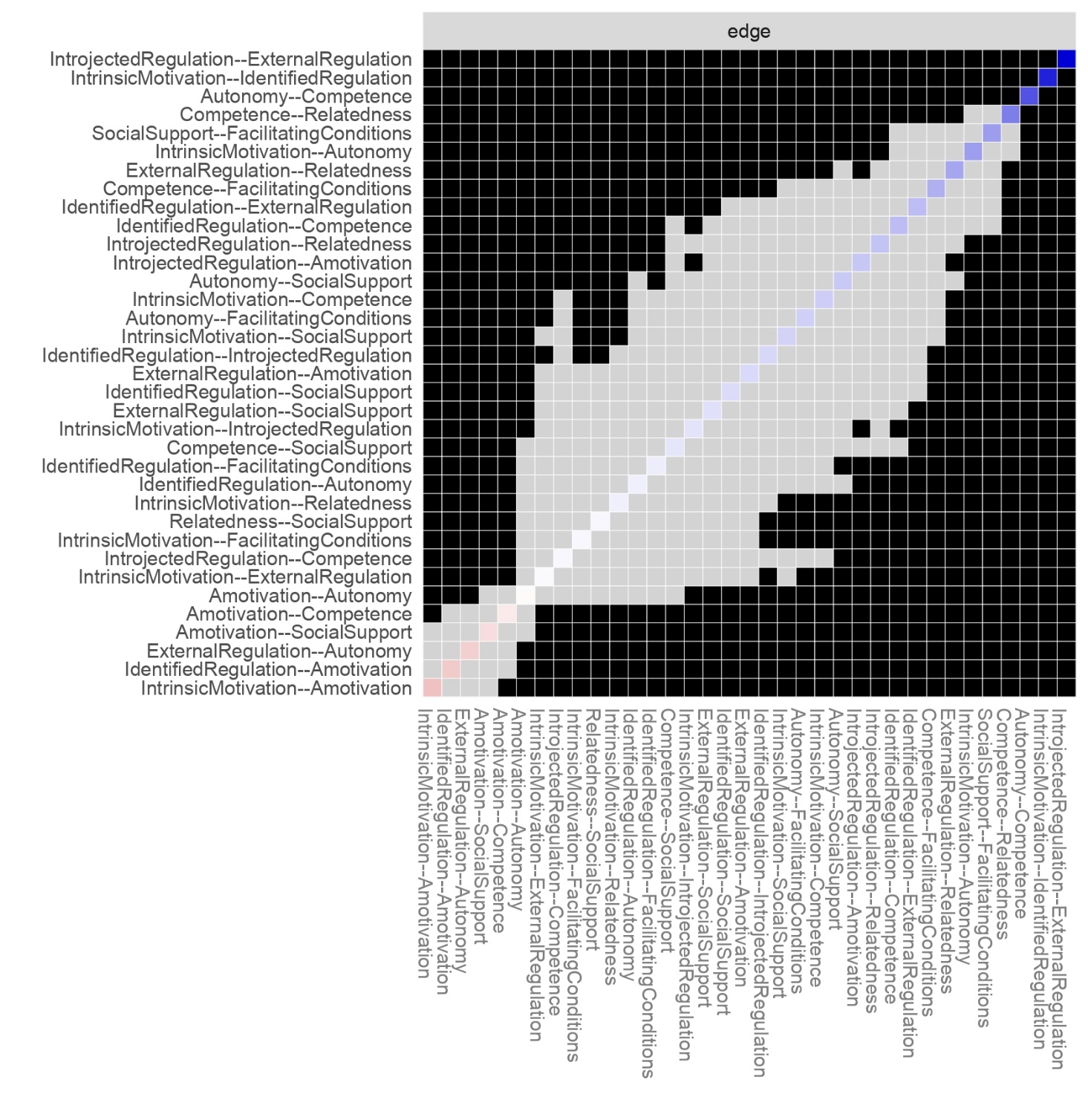


*Note.* This figure shows the node strength centrality difference for AI motivation and correlates. Black boxes denote nodes or edges that significantly differ from one another, while gray boxes indicate nodes or edges that do not, and the white box shows the value of node strength.

Supplementary Figure 6. Centrality stability for AI motivation and correlates

**
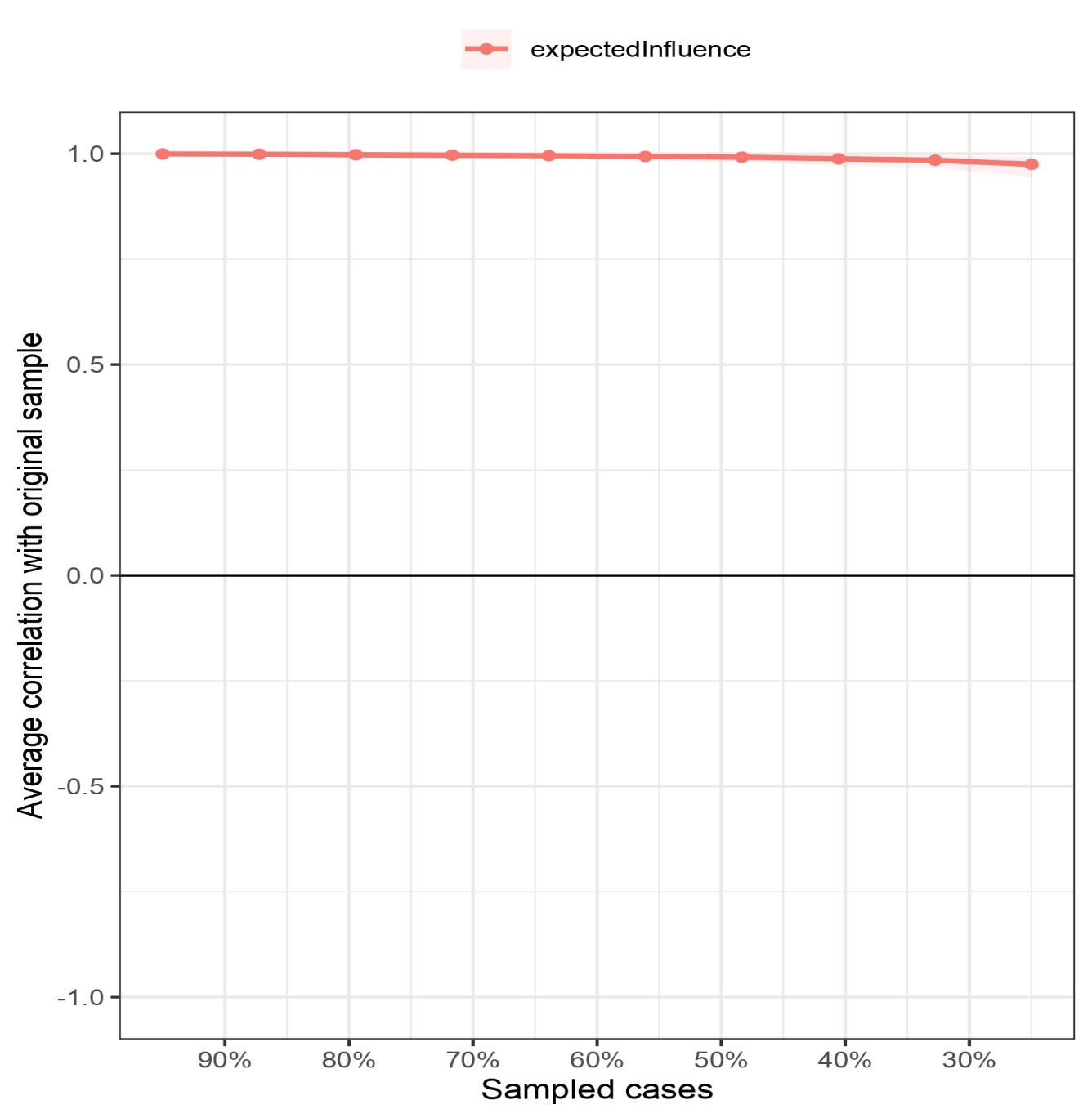
**

*Note.* This figure depicts the centrality stability for AI motivation and correlates. The x-axis illustrates the sample decrease from 95% to 25% of the original sample, and the y-axis illustrates the changes in correlation estimates between the subsample and the original entire sample. Lines indicate the means, and areas indicate the range from the 2.5th quantile to the 97.5th quantile.

**Section C**

Confirmatory factor analyses (CFA), structural equation modeling (SEM), and network analysis are employed to address distinct research questions. CFA is primarily used to validate the construct validity of the responses to the scale. SEM goes a step further by testing the theoretical linkages among latent constructs.

In contrast to CFA and SEM, network analysis (1) conceptualizes constructs as interconnected systems of elements and (2) generates an undirected graph, estimating edges between all nodes. The network analysis emphasizes the dynamic interplay among all variables rather than viewing them in isolation. More importantly, the network analysis approach enables the identification of system components (network nodes) and the strength of the connections between them (network edges), offering valuable insights for determining impactful intervention targets.

Given the prevalence of CFA and SEM, we conducted these analyses as a supplement to the network analysis. The results may be particularly relevant for those interested in the validity of the measurement scales or the relationships among the constructs.

Figures 3.1 and 3.2 present the results of CFAs. One model is a five-factor model encompassing distinct types of AI motivation and another one is a mass [CFA](https://www.sciencedirect.com/topics/social-sciences/confirmatory-factor-analysis) containing all constructs. Both models had satisfactory model-data fit indices.

Figure 3.3 shows the SEM results. Before testing a hypothesized model, researchers must define the paths and directions of relationships. For instance, some researchers may choose to examine the predictive effects of basic needs satisfaction on different types of motivation, while others may focus on evaluating the impact of motivation on basic needs satisfaction. Once this decision is made, alternative possibilities are typically excluded from consideration. As shown in the figure, we evaluated how different types of AI motivation were shaped by correlates. We can conclude that the basic needs satisfaction, social support, and facilitating conditions were significantly associated with the five types of motivation, albeit with varying effect sizes; however, we cannot ascertain which factor is the most central in the network.

Supplementary Figure 7. A five-factor model encompassing distinct types of AI motivation

**
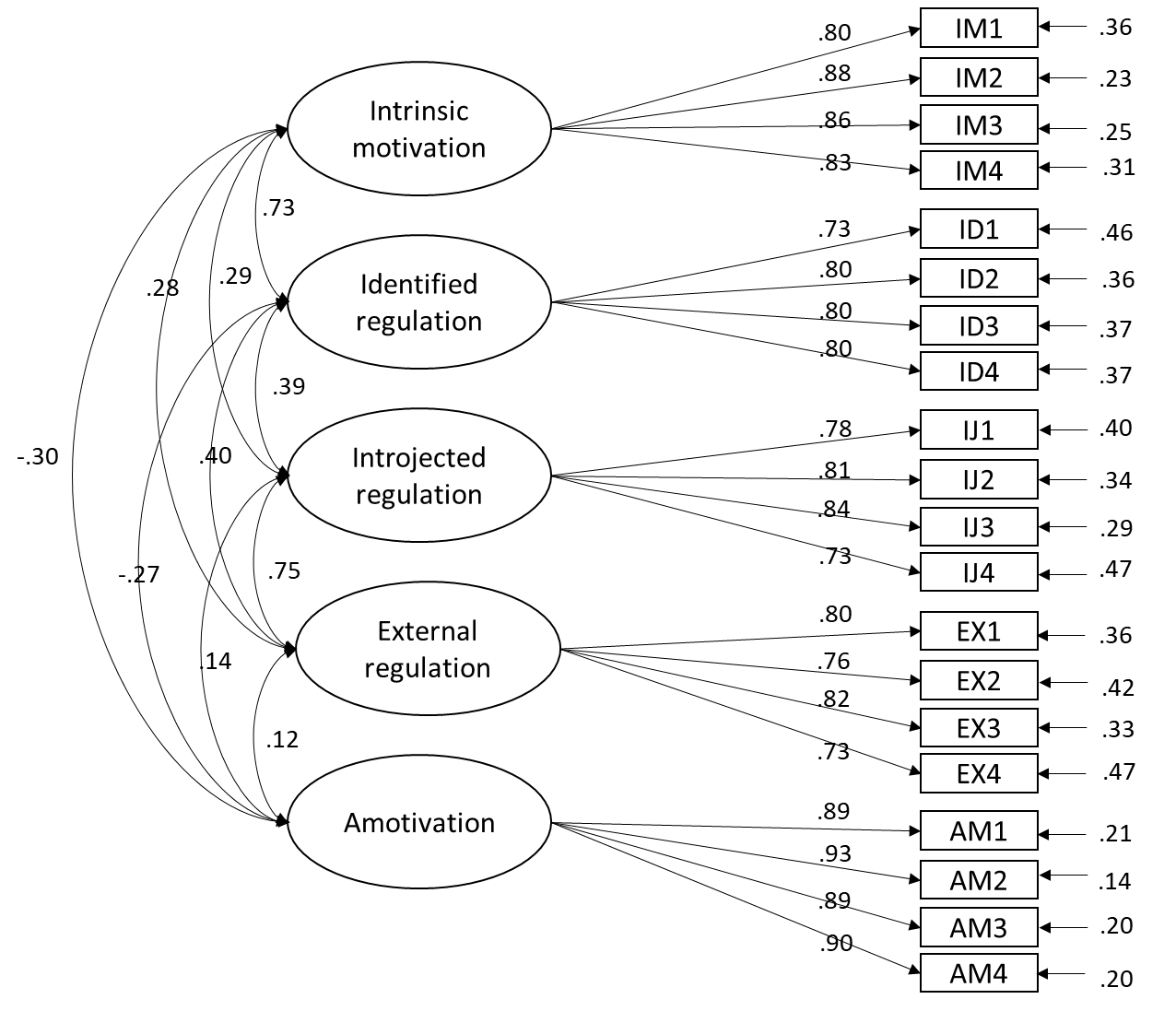
**

***Note.*** Results of CFA showed satisfactory model-data fit: CFI = .955, TLI = .946, RMSEA=.063 [.059, .066], SRMR = .044.

Supplementary Figure 8. A mass [CFA](https://www.sciencedirect.com/topics/social-sciences/confirmatory-factor-analysis) containing all constructs

**
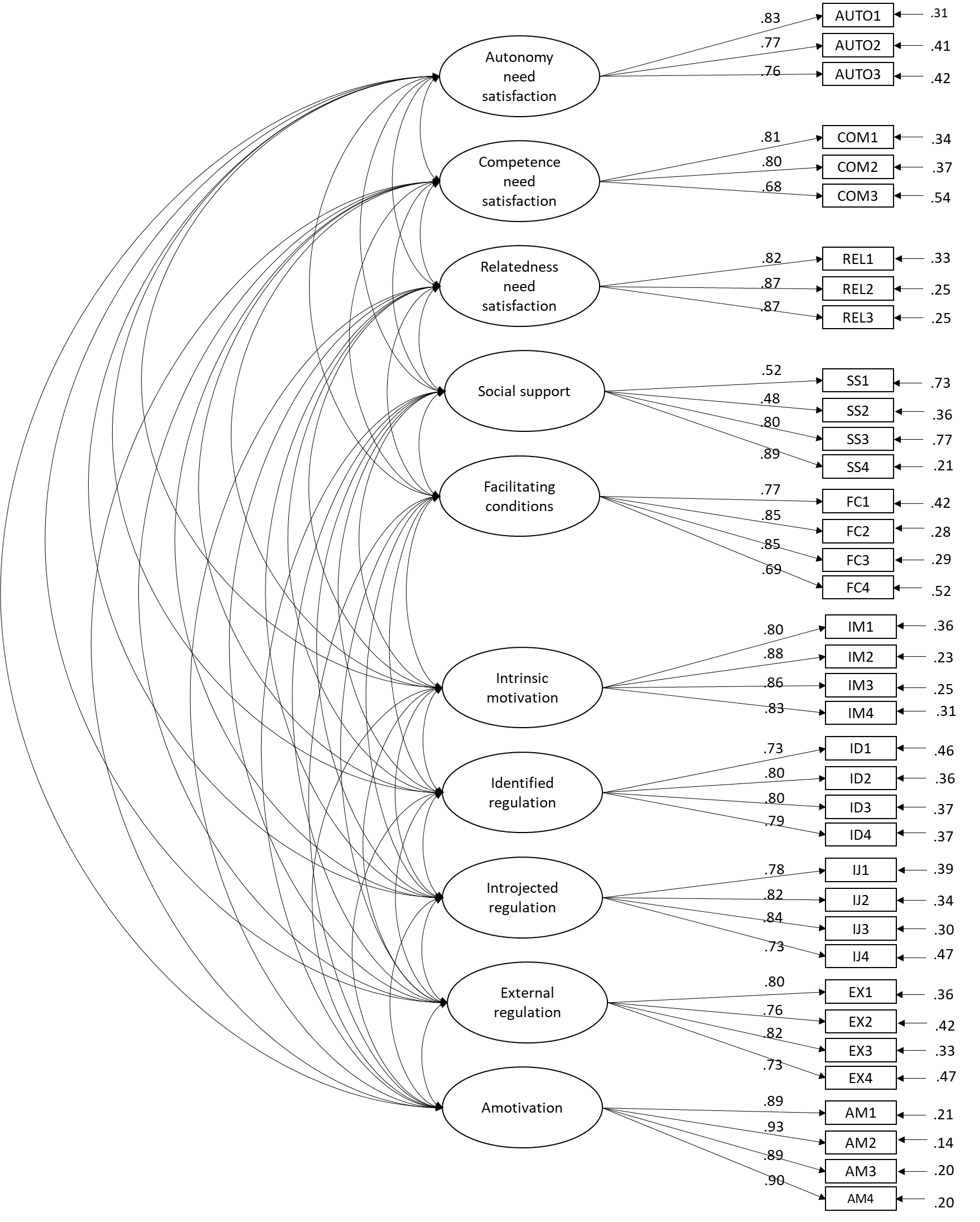
**

***Note.*** Results of confirmatory factor analysis showed satisfactory model-data fit: CFI = .931, TLI = .921, RMSEA=.052 [.050, .054], SRMR = .043; The figure omitted the correlational coefficients among variables for the sake of parsimony.

Supplementary Figure 9. The relationship between different types of motivation to learn AI and their correlates

**
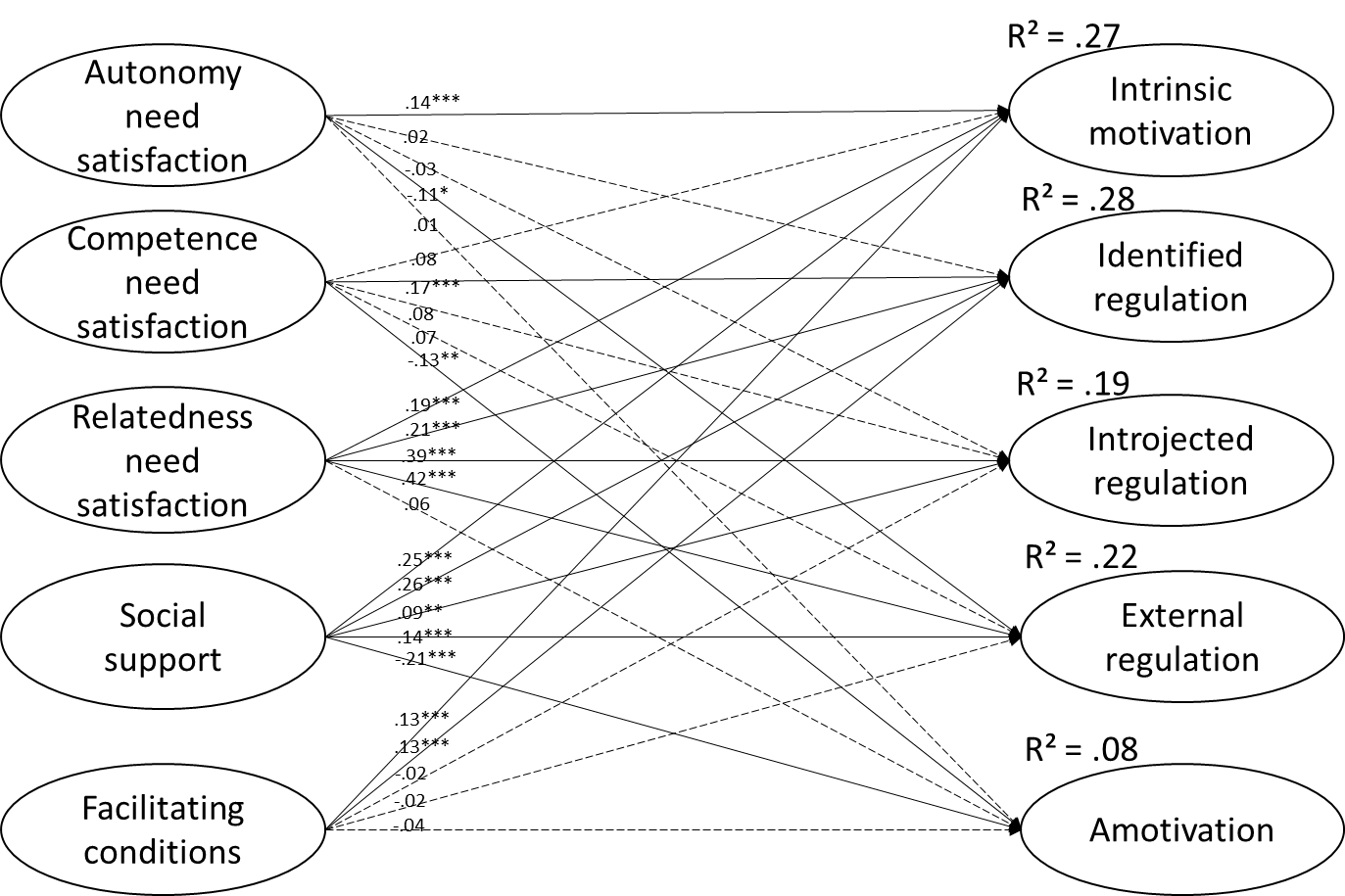
**

*Note.* Results of structural equation modeling showed satisfactory model-data fit: CFI = .931, TLI = .921, RMSEA=.052 [.050, .054], SRMR = .043.

**Section D**

This section includes items measuring motivation, need satisfaction, social support, facilitating conditions, engagement, AI literacy, and AI competency.

Supplementary Table 2. Items for variables of interest

| **1. Motivation to learn AI** |
| --- |
| **Intrinsic motivation** |
| I enjoy learning with AI very much. |
| Learning with AI is fun for me. |
| I feel satisfied through learning with AI. |
| Exploring knowledge with AI is fascinating to me. |
| **Identified regulation** |
| Learning with AI could enhance my effectiveness. |
| Learning with AI is important for my academic performance. |
| Learning with AI is important for my future career. |
| Learning with AI is important in helping me deal with future changes. |
| **Introjected regulation** |
| I will feel guilty if I don’t learn with AI. |
| I feel a sense of obligation to learn with AI because others expect it of me. |
| I will feel ashamed if I cannot learn with AI. |
| I will feel ashamed if others think I am incapable of learning with AI. |
| **External regulation** |
| I have no other choice but to learn with AI. |
| Learning with AI is one of the rules. |
| I will get in trouble if I don’t learn with AI. |
| I’m required to learn with AI. |
| **Amotivation** |
| Learning with AI is meaningless. |
| I cannot come to see why I need to learn with AI. |
| I don’t know what I am getting out of learning with AI. |
| Learning with AI is wasting my time. |
| **2.** **Basic psychological needs satisfaction** |
| **Perceived autonomy need satisfaction** |
| I feel like I can make a lot of input in deciding how I use AI in learning. |
| I feel a sense of freedom when using AI. |
| I have many opportunities with the AI to decide for myself how to learn. |
| **Perceived competence need satisfaction** |
| I think I am pretty good at learning with AI. |
| I have been able to learn interesting new knowledge with the AI. |
| I feel a sense of accomplishment from learning with the AI. |
| **Perceived relatedness need satisfaction** |
| When I learn with AI, I feel connected with my teachers. |
| When I learn with AI, I feel connected with my classmates. |
| When I learn with AI, I feel a sense of belonging in the class. |
| **3. Social support** |
| My school encourages the use of AI. |
| My teachers allow us to use AI for learning. |
| My classmates feel that it is important to learn with AI. |
| My friends feel that it is important to learn with AI. |
| **4. Facilitating conditions** |
| I can gain access to information about AI easily. |
| I can continuously improve my AI knowledge from many open sources. |
| I can easily find help when I need to know more about AI technology. |
| I can download many AI applications to test their efficacy. |
